# Workforce, regulation and capacity needed for integration of traditional medicine

**DOI:** 10.2471/BLT.25.293560

**Published:** 2025-10-23

**Authors:** Vivian Lin, Rachel Canaway, Anne-Louise Carlton, Nadine Ijaz, Gupteswar Patel, Natewinde Sawadogo, Hongguo Rong, Kabir Sheikh

**Affiliations:** a Faculty of Medicine, University of Hong Kong, Hong Kong Special Administrative Region, China.; b Department of General Practice & Primary Care, Level 4, Medical Building North Wing, Grattan St, University of Melbourne, 3010, Victoria, Australia.; c School of Health and Biomedical Sciences, RMIT University, Melbourne, Australia.; d Department of Law and Legal Studies, Carlton University, Ottawa, Canada.; e School of Health and Care Sciences, University of Lincoln, Lincoln, England.; f Institut Universitaire de Formations Initiale et Continue, Université Thomas Sankara, Ouagadougou, Burkina Faso.; g Centre for Evidence-Based Chinese Medicine, Beijing University of Chinese Medicine, Beijing, China.; h Global Business School for Health, Faculty of Population Health Sciences, University College London, London, England.

## Abstract

The widespread use of traditional, complementary and integrative medicines (traditional medicine) across the world suggests that integration of traditional medicine into the formal health system is one strategy for extending universal health coverage (UHC). To improve access to and the quality of traditional medicine services will require attention to strengthening the traditional medicine workforce. The challenges associated with making such improvements should not be underestimated due to the many different practices, service delivery models and education systems for traditional medicine, as well as relevant policy and governance frameworks. Countries have adopted varying strategies to integrate traditional medicine into health systems to date. We consider how to strengthen and build capacity of the traditional medicine workforce so it might better contribute to the UHC agenda. We examine key issues and challenges for traditional medicine, and suggest analytical models for understanding the complexity inherent to integration of traditional medicine and making sense of different components of the traditional medicine workforce.

## Résumé

L’utilisation généralisée de la médecine traditionnelle, complémentaire et intégrative (abrégée en « médecine traditionnelle ») à travers le monde suggère que son intégration dans le système de santé officiel est l’une des stratégies permettant d’étendre la couverture sanitaire universelle (CSU). Pour améliorer l’accès aux services de médecine traditionnelle et leur qualité, il faudra veiller à renforcer le personnel travaillant dans ce domaine. Il ne faut pas sous-estimer les défis liés à ces améliorations en raison de la grande diversité des pratiques, modèles de prestation de services et systèmes éducatifs en matière de médecine traditionnelle, ainsi que des cadres politiques et de gouvernance pertinents. À ce jour, des pays ont adopté des stratégies variées pour intégrer la médecine traditionnelle à leurs systèmes de santé. Le présent article examine comment renforcer et élaborer les capacités du personnel œuvrant dans la médecine traditionnelle afin qu’il puisse mieux contribuer aux objectifs de la CSU. Nous y examinons les questions et défis principaux pour la médecine traditionnelle et proposons des modèles analytiques permettant de comprendre la complexité inhérente à l’intégration de la médecine traditionnelle et de donner un sens aux différentes composantes du personnel travaillant dans ce domaine.

## Resumen

El uso generalizado de las medicinas tradicionales, complementarias e integrativas (medicina tradicional) en todo el mundo sugiere que la integración de la medicina tradicional en el sistema formal de la salud constituye una estrategia para ampliar la cobertura sanitaria universal (CSU). Mejorar el acceso y la calidad de los servicios de medicina tradicional requerirá prestar atención al fortalecimiento del personal sanitario de este ámbito. No deben subestimarse los desafíos que implica dicho fortalecimiento, dado el gran número de prácticas, modelos de prestación de servicios y sistemas educativos asociados a la medicina tradicional, así como los marcos normativos y de gobernanza pertinentes. Hasta la fecha, los países han adoptado diversas estrategias para integrar la medicina tradicional en sus sistemas de salud. En este artículo, se analiza cómo reforzar y desarrollar la capacidad del personal sanitario dedicado a la medicina tradicional, de modo que pueda contribuir mejor a la agenda de la CSU. Asimismo, se examinan las cuestiones y desafíos fundamentales para la medicina tradicional y se proponen modelos analíticos que facilitan la comprensión de la complejidad inherente a su integración y de los diferentes componentes de su personal sanitario.

## ملخص

إن الاستخدام واسع النطاق للطب التقليدي والتكميلي والمتكامل (الطب التقليدي) في جميع أنحاء العالم، يشير إلى أن دمج الطب التقليدي في النظام الصحي الرسمي يُعدّ إحدى الاستراتيجيات لتوسيع نطاق التغطية الصحية الشاملة. لتحسين الحصول على خدمات الطب التقليدي وتحسين جودتها، يتطلب ذلك التركيز على تعزيز القوى العاملة في الطب التقليدي. ولا ينبغي التهوين من التحديات التي تواجه تحقيق هذه التحسينات، نظرًا لاختلاف الممارسات، ونماذج تقديم الخدمات، وأنظمة التعليم في مجال الطب التقليدي، فضلاً عن أطر عمل السياسات والحوكمة ذات الصلة. وقد اعتمدت الدول استراتيجيات متباينة لدمج الطب التقليدي في النظم الصحية حتى الآن. ونحن ندرس كيفية تعزيز وبناء قدرات القوى العاملة في الطب التقليدي، حتى تُسهم بشكل أفضل في تحقيق أجندة عمل التغطية الصحية الشاملة. كما ندرس القضايا والتحديات الرئيسية التي تواجه الطب التقليدي، ونقترح نماذج تحليلية لفهم التعقيد الكامن في دمج الطب التقليدي وفهم مختلف مكونات القوى العاملة للطب التقليدي.

## 摘要

传统、补充和整合医学（传统医学）在世界各地的广泛使用表明，将传统医学整合至正规卫生系统是有助于扩大全民健康覆盖 (UHC) 范围的一项策略。为了提升传统医学服务的可及性和质量，需要着重发展传统医学卫生人员队伍。由于各国在传统医学相关实践、服务提供模式、教育系统以及相关政策和治理框架方面存在巨大差异，因此，在提升此类服务的可及性和质量过程中面临的这些挑战不容轻视。迄今为止，为了将传统医学整合至卫生系统，各国已推行了各种策略。我们考虑了如何加强和推行传统医学卫生人员队伍的能力建设，以便其能更好地推进 UHC 议程。我们研究了在推行传统医学方面会遇到的关键问题和挑战，并提出了分析模型，以期了解传统医学整合过程固有的复杂性以及传统医学卫生人员队伍的不同组成部分。

## Резюме

Широкое использование традиционной, комплементарной и интегративной медицины (традиционной медицины) во всем мире позволяет предположить, что интеграция традиционной медицины в официальные системы здравоохранения является одной из стратегий расширения всеобщего охвата услугами здравоохранения (universal health coverage, UHC). Улучшение доступа к услугам традиционной медицины и повышение их качества потребует усилий для укрепления кадровых ресурсов традиционной медицины. Не следует недооценивать сложности на этом пути, так как в традиционной медицине существует множество различных практик, моделей оказания услуг и систем обучения традиционной медицине; различаются между собой также политики и рамочные стратегии на уровне правительств. В настоящее время в странах по-разному подходят к интеграции традиционной медицины в системы здравоохранения. Авторы рассматривают вопрос укрепления и развития возможностей работников традиционной медицины с тем, чтобы они лучше отвечали насущным задачам UHC. В статье рассмотрены основные проблемы и сложные аспекты, связанные с традиционной медициной, а также предлагаются аналитические модели для осмысления сложностей интеграции традиционной медицины и понимания различных компонентов кадровых ресурсов, присущих данной сфере.

## Introduction

Traditional medicine includes a diverse range of health-care practices used for the maintenance of health, and the prevention, diagnosis and management of illness.^1^ Rooted in cultural, historical and philosophical contexts, traditional medicine represents the “sum total of knowledge, skill, and practices based on the theories, beliefs and experiences indigenous to different cultures, whether explicable or not.”^2^ The term encompasses whole medical systems such as traditional Chinese medicine, Ayurveda, naturopathy, homeopathy and Indigenous healing systems.^3^ For some people or communities, traditional medicine is the preferred or most accessible type of health care.^4^^,^^5^

While biomedicine typically dominates, some health systems adopt traditional medicine as so-called alternative or complementary medicine,^6^ sometimes integrating it with mainstream care; for example, chiropractic, osteopathy, massage and acupuncture. When offered together with biomedicine, the umbrella term traditional, complementary and integrative medicine is used.^1^^,^^7^ This paper refers mainly to traditional and complementary medicine: for brevity, we use the term traditional medicine to refer to both.

The use of traditional medicine is reported by 170 Member States of the World Health Organization (WHO) and nearly half of the population in many high-income countries.^1^^,^^8^^,^^9^ Traditional medicine is part of primary health care in most countries.^1^ Evidence exists of increasing traditional medicine use,^10^ which is attributed to various factors including cultural preferences, dissatisfaction with conventional biomedicine, lack of biomedical services and a desire for more holistic, preventive approaches to health care.^11^^,^^12^ In some instances, use of traditional medicine has led to improved, cost-effective, long-term health outcomes.^3^^,^^13^

Globally, the goal to achieve universal health coverage (UHC) by 2030 does not currently look achievable.^14^ Given the prevalence of traditional medicine use in primary health care and the economic benefits forecast from a so-called whole-person health approach to care,^13^ adopting greater country-level standardization and improving traditional medicine quality could contribute to achieving UHC.^15^ A starting point for increasing this contribution is for governments to consider the adequacy of the traditional medicine workforce, education, regulation, financing and service-delivery systems. However, wide differences exist in system-level support for and acceptance of traditional medicine.^1^^,^^15^ For many countries, little or no documentation is available on how the traditional medicine workforce is constituted, what disciplines and therapeutic practices are offered, the educational standards and competencies required to practise safely and competently, or any occupational regulation or financing policy or strategy.^1^ Maximizing the potential of traditional medicine requires concerted policy effort to incorporate the traditional medicine workforce into the health-care and education systems.

In this article, we consider how to strengthen and build capacity of the traditional medicine workforce to better contribute to the UHC agenda. We examine key issues and challenges and suggest an analytical model for mapping the positioning of the traditional medicine workforce to develop pathways for its integration.

## Complexities of the workforce

To develop policies on the traditional medicine workforce requires an understanding of its complexities with regard to training, therapeutic systems and service delivery, and how these elements, as applicable to traditional medicine, relate to the rest of the health-care system. In the following sections we describe a framework for considering the complexities of the traditional medicine workforce and practice at the country level. Box 1 summarizes questions that can be asked by countries to deepen their understanding of the complex contextual factors underpinning the needs of the traditional medicine workforce, and the regulatory challenges and capacity-building. Mapping a country’s traditional medicine occupations, therapeutic practices, education programmes and institutions against each of the dimensions in Box 1 is a starting point for developing policy goals and strategies to more effectively incorporate traditional medicine into existing health-care delivery systems, workforce regulations, and education and training frameworks.

Box 1 **Understanding the complexities in the traditional medicine landscape**

***Complexity 1. Therapeutic systems and practices***

- Origin: are traditional medicine practices rooted in the ethnomedicine of specific communities or cultural groups?
- Knowledge transmission: are traditional medicine therapeutic practices codified in written sources or orally transmitted?
- Scope: do traditional medicine therapeutic practices constitute a comprehensive medical system, or do they consist of microsystems or stand-alone practices, products or devices?
- Therapeutic model: are the underlying philosophy and principles of the traditional medicine system oriented towards mechanistic or vitalistic, reductionist or holistic, and pathogenic or salutogenic understandings of health and disease?^16^

***Complexity 2. Service delivery***

- Form of recognition: is the expertise of traditional medicine practitioners affirmed through professional mechanism (e.g. licensing/statutory registration), non-licensing credentialing mechanisms (i.e. official recognition without statutory or government licence) or community-based recognition pathways?^17^
- Degree of recognition: are traditional medicine practitioners recognized by institutions (e.g. governments, funding bodies and research bodies) and are they working in the state-organized public or private sectors, or in the not-for-profit sector, or do they practise informally, responding to service requests by patients and communities?
- Practice context: is the work setting and payment structure for traditional medicine practitioners public or private?
- Extent of integration: how are traditional medicine practitioners included in the health system and do they experience exclusion, limitations, co-option and/or assimilation of their therapeutic practices?

***Complexity 3. Workforce training***

- Level and type of training: is training for traditional medicine practitioners available only for primary care generalists or also specialists, educators, researchers and/or community members?^17^
- Financing of educational programmes: are traditional medicine training programmes available only in the private sector, or also publicly funded, or mixed?
- Delivery of education: is traditional medicine training delivered principally by apprenticeship or mentorship and/or offered in more formal educational settings (e.g. post-secondary colleges, universities and non-profit or for-profit organizations)?
- Quality assurance: are traditional medicine education programmes subject to government, community-based and/or industry accreditation systems, or is there no external accreditation?

***Complexity 4. Health system contexts***

- Regulatory policy: is there regulation of traditional medicine practitioners according to their occupation and type of traditional medicine they practise; if so, what type/s, for example, voluntary association certification, government co-regulation, negative licensing and statutory registration?
- Products, practices and devices: are traditional medicines included under national medicines regulations or does no regulatory oversight exist?^18^
- Payment: is payment for traditional medicine services mostly out-of-pocket by service users or is there insurance coverage or tax-based financing?
- Service delivery experience: are traditional medicine services delivered in parallel with biomedical services, in collaboration with other health workers, or are fully integrated providers and service delivery models in place that offer both traditional medicine and biomedicine?^19^^,^^20^

Note: Traditional medicine here encompasses traditional, complementary and integrative medicine.

### Complexity 1: therapeutic systems and practices

Traditional medicine practices differ substantially between countries, which have unique combinations of therapeutic systems, bodies of knowledge and practices.^1^ The degrees of co-existence of different traditional medicine practices with conventional medicine also differ, as do the therapeutic models that inform practitioner skills and practices (see Box 1). Mapping different traditional medicine systems against the four dimensions – origin, knowledge transmission, scope and therapeutic model (Box 1) – should consider historical and cultural factors.^15^^,^^16^

### Complexity 2: service delivery

Traditional medicine practitioners operate in many different parts of the health system, with different degrees of integration into the biomedical service delivery systems. Mapping traditional medicine service delivery should consider at least four dimensions: form of recognition, degree of recognition, practice context and extent of integration (Box 1).^16^^,^^21^

### Complexity 3: workforce training

An added complexity of positioning the traditional medicine workforce within the health system is the variety of ways that practitioners are trained, including within the same traditional medicine occupation. Educational arrangements and qualifications related to the traditional medicine workforce for different traditional medicine occupations may be mapped against the four dimensions: level and type of training; financing of education programmes; delivery of education; and quality assurance (Box 1). 

### Complexity 4: health system contexts

If UHC is the main objective for providing all people with access to good quality health services without financial hardship, and if strengthening traditional medicine can contribute to achieving UHC, then understanding how the traditional medicine workforce can be better integrated into health-care systems is key. At present, the extent to which such integration is occurring varies, both across countries and across professions within countries. Integration may occur at the level of policy, service delivery and the service user.^7^^,^^15^ Overall, policies shaping health systems may be described as exclusionary, tolerant, inclusive or integrated with regard to their level of acceptance of traditional medicine. A tolerant system “is based entirely on allopathic medicine, but some [traditional medicine] practices are tolerated by law”; an inclusive system recognizes traditional medicine “but has not yet fully integrated it into all aspects of health care”; and an integrated system officially recognizes and incorporates traditional medicine “into all areas of health-care provision.”^22^ Health system contexts may be mapped against the four dimensions: regulatory policy; products, practices and devices; payment; and service delivery experience (Box 1).

## Integration and the workforce 

A systematic review of factors influencing integration of traditional and mainstream medicines suggests that policy, education and trust are the most important obstacles, as well as enablers, for traditional medicine integration at all levels of the health system.^23^ Whether national traditional medicine policies exist or not, different practices, services or practitioners may receive different degrees of policy attention. Policies can also be interpreted in different ways, in some instances with the traditional medicine workforce being co-opted or directed to provide biomedical services; for example, doctors of Ayurveda or traditional Chinese medicine who receive training in biomedical science in addition to traditional methods and philosophies.^24^^,^^25^ Within any health-care system, multiple levels and types of integration can apply to different traditional medicine occupations, which reflects a wide range of contextual factors, including history, culture, ideology, politics and economics.^15^ Mapping these dimensions can help set realistic policy goals for traditional medicine integration.

The extent and nature of integration of various traditional medicine practitioners within a health-care system may be mapped against the dimensions outlined in Box 1.

A person-centred approach aims to shift the system from parallel service delivery, where consumers act as the interface between providers, to integration of both traditional medicine and biomedicine at the point of delivery of clinical care.^7^^,^^19^ At present, however, truly integrated health services and systems are few. The policy system, whether tolerant, inclusive or integrative (as described earlier), determines the service delivery models (parallel practice, co-location, cross-referral or integration),^19^ which hence determines whether patients experience fragmented, partially coordinated or integrated care. As policies become more integrative, service delivery becomes more collaborative, thus enabling patients to benefit from a unified system rather than navigating fragmented systems on their own.^7^

Despite the complexities and variations, it is the service users (patients and families) who choose the kind of care that best manages their health-care needs, in line with preferences, cultural specificities, accessibility and affordability,^5^ especially since out-of-pocket health-care costs are worsening in most countries.^14^ Educational initiatives such as the Informed Health Choice project are helping communities across many countries think critically about health, health claims and choices relevant to traditional medicine and biomedicine.^26^

## Policy on workforce strengthening

If UHC is defined as ensuring access to good quality services without undue financial hardship, then the key policy objectives relating to the traditional medicine workforce are to: (i) understand key features of the traditional medicine workforce, service user demand and service delivery models; (ii) ensure the quality, safety and benefit of the services traditional medicine practitioners provide; and (iii) increase appropriate and equitable access to effective traditional medicine services.

Systematically addressing these objectives should contribute to understanding the regulatory practice and access gaps,^27^ and strengthen public and institutional trust in traditional medicine providers.

## Challenges

The integration of traditional medicine into biomedical health-care systems raises concerns about potential misappropriation, subjugation, and co-option of traditional medicine practitioners and their associated therapeutic knowledges and practices.^25^ While most traditional medicine disciplines include foundational biomedical education, biomedical health workers are not normally required to learn about traditional medicine disciplines, although traditional medicine training for students of biomedicine is increasing.^28^^,^^29^ Ideally, integration policies should explicitly recognize the cultural roots and traditions of autonomous traditional medicine practice.^30^ Addressing the challenges of integrating traditional medicine requires consideration of the broader social and cultural dimensions related to the practice (Box 1), beyond the immediate political and economic implications within policy development.^15^^,^^31^

### Practitioner level

Policy and planning related to traditional medicine at the practitioner level requires attention to workforce competency, training and regulation.

#### Competency assurance and regulation

Minimum qualification and practice standards need to be set for service provision to prevent harm and ensure ongoing access to culturally relevant health care. Such standards are fundamental to assuring traditional medicine practitioner competency, especially for occupations whose therapeutic practices pose potential risks to public health and safety.

Health workforce regulation varies from country to country,^32^ including whether health workforce regulations are specific to traditional medicine occupations or are more generic.^1^^,^^18^ Many types of occupational regulation and governance have been applied to traditional medicine occupations and practitioners, but many policy challenges exist such as accreditation standards, administration of licensing examinations and determination of scopes of practice.^33^^,^^34^

To address these challenges, statutory registration of traditional medicine occupations would allow for effective management of the health labour market.^35^^,^^36^ Four main types of occupational regulation have been described: voluntary certification (by profession-led bodies); government and/or profession co-regulation; negative licensing (i.e. imposing restrictions on a licensee, including suspension); and statutory registration (or occupational licensing).^18^ Statutory registration has been growing rapidly for traditional medicine occupations, often to preserve Indigenous medicine traditions in low- and middle-income countries and in response to pressure from representative bodies, such as professional associations of traditional medicine practitioners, in high-income countries.^33^ Generally, more stringent regulatory oversight is needed for traditional medicine occupations with a higher risk profile, such as practices that use ingestive therapies, skin penetration and manipulative therapies.^18^

#### Training standards and validation

Validated, minimum standards need to be set for traditional medicine education for students of traditional medicine and of biomedicine.

Education for traditional medicine practitioners varies from informal apprenticeships to postgraduate qualifications, and from oral transmission of knowledge to programmes in private colleges and publicly funded higher education institutions.^37^ Achieving consensus on what constitutes an adequate education for safe and competent practice is a challenge.^38^ Furthermore, there is little uptake of traditional medicine education among biomedically trained health workers.^28^ Given the cultural specificities that characterize the traditional medicine field, global standardization of educational requirements may not account sufficiently for local contextual requirements.^30^ Even where accredited programmes exist within formal educational institutions, debate persists about the inclusion of biomedical sciences, interprofessional education and new pedagogical approaches to teaching (for example, simulation, problem-based learning and international electives).^28^^,^^36^^,^^39^

To address these challenges, national qualification frameworks, competency-based training and government-led accreditation standards and processes can assure the quality of traditional medicine education and training for traditional medicine occupations and biomedical providers.^28^ The increasing use of digital technologies and learning via electronic media within health profession education is an opportunity for modernization of the methods used in traditional medicine education.^40^

#### Balancing tradition and modernity

The application of traditional knowledge in health-care practice needs to be reconciled with modern health-care demands.

However, reconciling traditional knowledge with modern health care presents fundamental challenges for traditional medicine education and governance.^36^^,^^41^ Tensions may exist between the desire to preserve and transmit cultural values and classical approaches through the generations of healers and to patients, and the desire of traditional medicine practitioners to have institutionally-sanctioned status and recognition alongside their biomedically trained colleagues.^38^^,^^41^

Policies should therefore be developed to encourage harmonious integration of traditional medicine and biomedicine or intercultural health-system models. It is also important to foster environments conducive to mutual education, reciprocal respect, synchronized progress and the best use of respective strengths. Ideally, integration policies should explicitly recognize the cultural roots and traditions of autonomous traditional medicine practice.^30^ For instance, since 1955 the *xi xue zhong* (west learning from east) initiative has encouraged biomedically trained practitioners to embark on studies of traditional Chinese medicine.^42^ In several Latin American countries, intercultural health system models that consider the world views of Indigenous Peoples have been implemented as a so-called decolonizing mechanism to advance more even power relations with biomedicine.^20^^,^^43^

### System level

Policy and planning at the health system level requires: (i) attention to workforce planning; (ii) decreased financial barriers to access; (iii) enablement of practitioners to access the tools of their trade (such as medicinal plants, oils, minerals), preparatory equipment, manual therapy tools (such as, acupuncture needles), *materia medica*, and educational or diagnostic texts; (iv) assurance of quality and safety of services (beyond individual practitioner competencies); (v) performance monitoring; (vi) advancement of the uptake of traditional medicine research evidence into policy and training of biomedical and traditional medicine practitioners; and (vii) increased respect and awareness between practitioners.

#### Workforce planning and data collection

Routinely collected data on the traditional medicine workforce and traditional medicine use should be used for planning and policy development.

Most countries do not routinely collect data on the health workforce supply and demand. Countries without systems to regulate occupations, or with few traditional medicine occupations (licensed or registered, for example, Australia) have incomplete data on the traditional medicine workforce. Therefore, they have to rely on one-off surveys or reporting by professional associations and/or educational programmes to estimate workforce size.^1^ Quantifying demand for traditional medicine services presents an even greater challenge, and also relies on one-off surveys or national health surveys to understand usage patterns and motivations.^9^

In a highly integrated system, such as China, detailed data are readily available, including the number of traditional Chinese medicine graduates and traditional practitioners, where they practise and when they are due to leave the workforce. In most countries, it is possible to consider how relevant questions might be incorporated into a variety of regular surveys, whether focused on population health, traditional medicine usage, or on social, economic or labour force status of traditional medicine practitioners.

#### Access and use

It is important to enable better access to and use of traditional medicine as part of a UHC strategy.

However, the use of traditional medicine services is often closely related to financing arrangements, and governance issues may undermine access and use.^44^ In many countries, traditional medicine is either an out-of-pocket expense or receives partial cover from private health insurance.^1^ In impoverished areas, traditional medicine practitioners may be part of an informal economy that operates without monetary compensation. An important question is how best to incentivize care planning, coordination and referrals between traditional medicine and biomedical health workers, regardless of the existing remuneration systems.

To address this challenge, specific studies need to be undertaken to develop payment and incentive systems appropriate to the overall health financing arrangements. In countries where traditional medicine practitioners are employed in the public sector, such as in China and Thailand, no significant financial barriers to access exist. Similarly, in India, traditional medicine practitioners are co-located in public primary health-care facilities and offer free services. Increasing access to good quality traditional medicine services also requires that traditional medicine practitioners can lawfully access the tools of their trade, such as medicinal plants.

#### Trust, quality and safety 

Traditional medicine quality, credibility and safe use must be assured through knowledge generation and sharing.

However, communities and individuals often use traditional medicine alongside pharmaceutical medicines and may not share this information with their respective practitioners. Beyond the effectiveness and efficiency of traditional medicine clinical care and service delivery, questions remain about the extent to which traditional medicine is contributing to better outcomes and a better health system. The importance of infrastructure and monitoring systems to assure quality and safety of traditional medicine products and services cannot be understated.

Therefore, for traditional medicine to gain and maintain credibility, trust in and connection to its holistic foundations, systems for research, monitoring and evaluation must be appropriate and respectful of the traditions in addition to providing good quality and trustworthy evidence.^45^^–^^47^ Additionally, systems to support translation of research findings into policy and practice are needed.^31^ Traditional medicine research, from clinical effectiveness to health service use and product development, contributes to service improvement and requires expertise in basic biomedical sciences.^4^ Schemes that support mainstream research need to allocate funding to develop traditional medicine research and scholarship.^4^ The RAND Corporation’s Research Across Complementary and Integrative Health Institutions (REACH) Center is one example of support for collaborative research to support multidisciplinary systems of health care.^48^

## Next steps: workforce models

How traditional medicine workforces are governed depends on the approaches that countries take to determine the place of traditional medicine in health systems, as well as on many political, social and economic considerations. Different approaches are evident.^15^ For example, in China, Japan and Republic of Korea, long-standing institutional arrangements are in place for education, clinical training, service delivery, research and product development.^37^^,^^49^ In several high-income English-speaking countries (such as Australia, Canada, New Zealand and the United States of America), there is statutory registration for some traditional medicine occupations, and established institutions exist for quality assurance of traditional medicine education programmes.^18^^,^^31^^,^^34^ Although traditional medicine is widely used in many African and Latin American countries, the policy on traditional medicine, and legal, financing and service delivery systems for it are mostly still under development.^43^^,^^50^ A strong government commitment to service integration can be seen in some middle-income countries, such as Brazil, India, Thailand and Viet Nam.^2^^,^^51^^,^^52^

Given the complexities outlined in our article, the analytic framework (summarized in Box 2) can be used to better describe and evaluate the features, strengths and challenges associated with different approaches to the governance of the traditional medicine workforce across jurisdictions. Mapping the positioning of the traditional medicine workforce in a comprehensive way can point to the variety of issues that need to be addressed in developing pathways towards integration.

Box 2 **Traditional medicine workforce analytic framework**

***Workforce training***

- Public post-secondary
- Private post-secondary
- Non-profit organization
- Corporate or private, for-profit organization
- Community-based^a^

***Training validation***

- Government
- Community organization
- Private or corporate
- Community norm or social acceptance^a^

***Workforce governance***

- Government licensing and/or registration
- Negative licensing
- Government and/or professional body co-regulation
- Non-statutory credentialing
- Community-based recognition^a^

***Financing and reimbursement***

- Government
- Insurance
- Patient
- Local economy (including exchange or barter economy)^a^

***Interaction with the dominant health-care system* *^7^* *^,^* *^22^***

- Exclusionary
- Tolerant
- Inclusive
- Integrative
- Intercultural health and therapeutic pluralism^b^

^a^ Community-based, local economy elements encompass Indigenous systems of traditional medicine.^b^ The intercultural model is a “conceptual avenue through which [traditional and complementary medicine has] made inroads into national health systems” and relates to the “equitable interaction of diverse cultures” which includes biomedical and traditional medicine systems with a parallel or horizontal relationship (rather than biomedical dominance). In cultures that embrace policies of therapeutic pluralism, constitutional mandates may exist to preserve traditional medicine as part of the preservation of Indigenous identity, culture and tradition.^20^^,^^43^

In countries where traditional medicine is well established and integrated within the mainstream health system, the drivers of successful governance of the traditional medicine workforce need to be better understood. They are likely to include some combination of political commitment, professional and community-based leadership, and research evidence.

## Conclusion

Effective multilevel integration of the traditional medicine workforce with mainstream health systems can contribute to achieving UHC. While strengthening the traditional medicine workforce to improve integration has many complex dimensions, assurance of traditional medicine quality and safety is the priority.

Integration strategies will vary from country to country, with policy and planning strategies that consider community preferences, availability, accessibility, acceptability and existing health services. Key starting points are to: (i) better understand traditional medicine providers, institutions and service users within their local contexts; and (ii) identify priority interventions to strengthen workforce planning, education, regulation, financing and service delivery, and increase trust, respect and awareness between biomedical and traditional medicine practitioners.

Workforce strengthening requires assessment of the risks, benefits and competencies needed for traditional medicine practices. A stepwise approach, tailored to specific service and workforce priorities and fitting with the overall UHC roadmap, could facilitate more effective integration.

There will be debate about government intervention in so-called modernizing traditional practices and whether integration is a good thing. Maintaining traditional knowledge and skills, if not teaching methods, is important to many communities. Fear about the loss or subjugation of cultural traditions in the face of a dominant biomedicine-based health system needs to be addressed thoughtfully. No universal best practice method exists for bringing traditional medicine into the UHC agenda, but lessons can be learnt from the variety of approaches seen in different countries.
